# Adrenal schwannoma: A case report of an unusual incidentaloma

**DOI:** 10.1016/j.ijscr.2021.106018

**Published:** 2021-05-26

**Authors:** Sujan Timilsina, Surya Prakash Joshi, Sujan Sharma, Sanjeev Kharel, Shovana Karki, Sansar Babu Tiwari, Durga Pandit, Purushottam Parajuli

**Affiliations:** a Maharajgunj Medical Campus, Institute of Medicine, Kathmandu, Nepal; b Department of Pathology, Tribhuvan University Teaching Hospital, Kathmandu, Nepal; c Department of Urology and Kidney Transplant Surgery, Tribhuvan University Teaching Hospital, Kathmandu, Nepal

**Keywords:** Incidentaloma, Retroperitoneal tumor, Schwannoma

## Abstract

**Introduction and importance:**

Adrenal schwannomas are extremely rare tumors often misdiagnosed. The patients are usually asymptomatic while some present with non-specific abdominal pain. Only a few cases are reported to date.

**Case presentation:**

We here present a case of a 55-year-old Nepalese man presented with nonspecific abdominal pain at our Outpatient Department (OPD) found to have mass on ultrasonography of abdomen. On further investigation with Contrast Enhanced Computerized Tomography (CECT) of the abdomen and pelvis, a well-defined heterogeneous adrenal mass of size (7.8 ∗ 8.3 ∗ 6) cm with foci of calcification was seen in the left retroperitoneum. The intraoperative finding of adrenal mass and histopathology of resected mass was suggestive of schwannoma arising from the adrenal gland which was further confirmed by immunohistochemistry.

**Clinical discussion:**

Adrenal schwannoma can mimic tumors like pheochromocytoma, adrenal adenoma, cortical carcinoma, neuroblastoma, and other masses. Only 1–3% of schwannomas are retroperitoneal. Radiological findings of this tumor are non-suggestive. The histological section shows spindle cells with Antoni A and Antoni B regions while positive staining of S-100 protein in Immunohistochemistry.

**Conclusion:**

The diagnosis of adrenal schwannoma in the retroperitoneum is often challenging. The treatment of choice is surgical resection with a good prognosis.

## Highlights

• Adrenal schwannomas are extremely rare tumors.
• These tumors are mostly found incidentally and often asymptomatic.
• Complete surgical resection of the tumor is a must for good prognosis.

## 1 Introduction

Schwannoma is benign nerve sheath neoplasms arising from the neural crest cells. They are slow-growing and uncommon [1,2]. Mostly, they originate from the central nervous system but sometimes also found in the pancreas, stomach, kidney, and liver [3]. 1–3% of all schwannoma are retroperitoneal with median age involvement at 49 years and a slight female dominance (male/female ratio 1:1.2) [4,5]. Schwannoma is difficult to differentiate from other tumors due to the absence of specific radiological and clinical features. Histopathological and immunohistochemical studies of surgically resected specimens are the primary method of reaching an absolute diagnosis [4,6].

Here we report the case of left adrenal schwannoma incidentally discovered in a 55-year-old man presented in OPD with non-specific abdominal pain.

This work has been reported in line with the SCARE criteria [7].

## 2 Case presentation

A 55-year Nepalese man, presented in our Out Patient Department with a chief complaint of non-specific central abdominal pain for six months. The abdominal pain was vague, generalized, mild intensity, non-radiating, and relieved by analgesics. He was a farmer by occupation and didn't have any prior surgical history and family history of malignancy. He didn't have a history of trauma, weight loss, fever, and abdominal distension.

On physical examination, he was afebrile and all vitals were stable. On per-abdomen examination, there was no tenderness and no palpable mass. The examination of all other systems was grossly intact. He had the habit of chewing tobacco regularly for the last 30 years and was a social drinker.

On blood investigations, hemoglobin level was found to be 11.6 g/dl, total leukocyte count (TLC) was 11,000/μl, urea level was 6.9 mmol/l, creatinine level was 125 μmol/l (normal range = 60–130 μmol/l), sodium level was 132 mEq/l (normal range = 135–145 mEq/l), potassium level was 3.7 mEq/l (normal range = 3.5–5.2 mEq/l). On 24-h urine examination, vanillylmandelic acid (VMA) level was 5.4 mg/24 h (normal range < 13.6 mg/24 h) and metanephrine level was 82.64 μg/24 h (normal range = 73–808 μg/24 h).

**Fig. 1:**
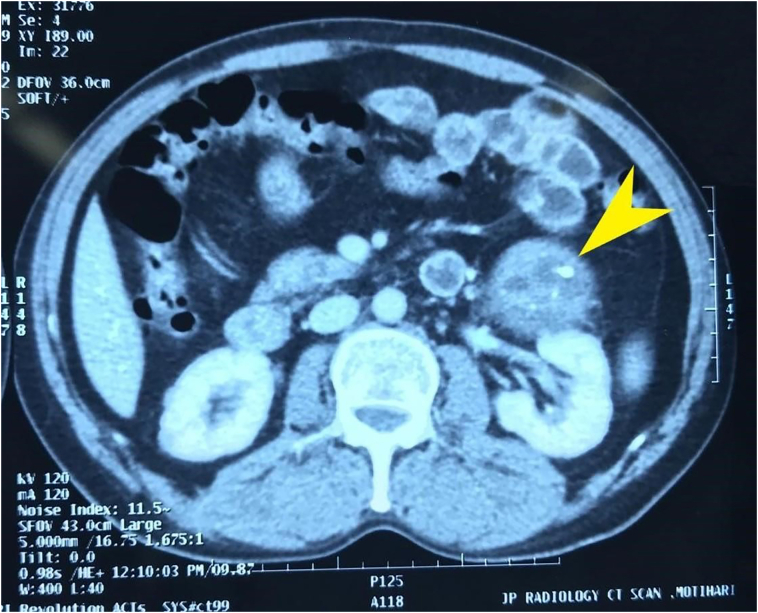
CECT of abdomen & pelvis showing a heterogeneous adrenal mass of size (7.8 ∗ 8.3 ∗ 6) cm with foci of calcification in the left retroperitoneum (yellow arrow).

The patient underwent ultrasonography (USG) of the abdomen and pelvis for evaluation of abdominal pain; which showed a heterogeneous mass near the left adrenal gland. For further characterization of mass, contrast-enhanced CT of the abdomen and pelvis was done which showed a well-defined heterogeneous adrenal mass of size (7.8 ∗ 8.3 ∗ 6) cm with foci of calcification in the left retroperitoneum as shown in Fig. 1. The case was discussed among surgeons' teams while the modality of treatment and risk of the procedure were explained to the patient. The team then finalized the decision to resect the tumor mass after the patient consent. Taking into account the size of the tumor and favorable prognosis, laparoscopic left adrenalectomy was planned which was converted to open adrenalectomy due to complications like adhesions and bleeding at the middle of the procedure. The adrenal mass was dissected from surrounding fibrous tissue and was removed after the ligation of the adrenal vein and artery. The hemostasis was secured and then drain was placed in the left perirenal area. The resected mass was sent for histopathological examination.

**Fig. 2:**
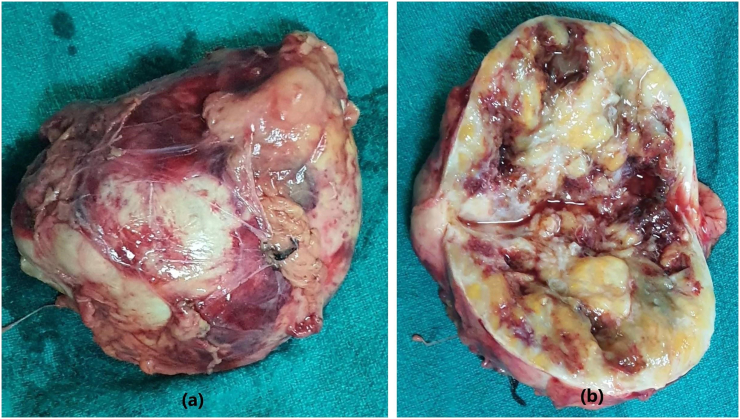
(a, b): Gross appearance of the resected adrenal gland shows well-encapsulated mass which on the cut section shows heterogeneous gelatinous to solid grey white areas.

**Fig. 3:**
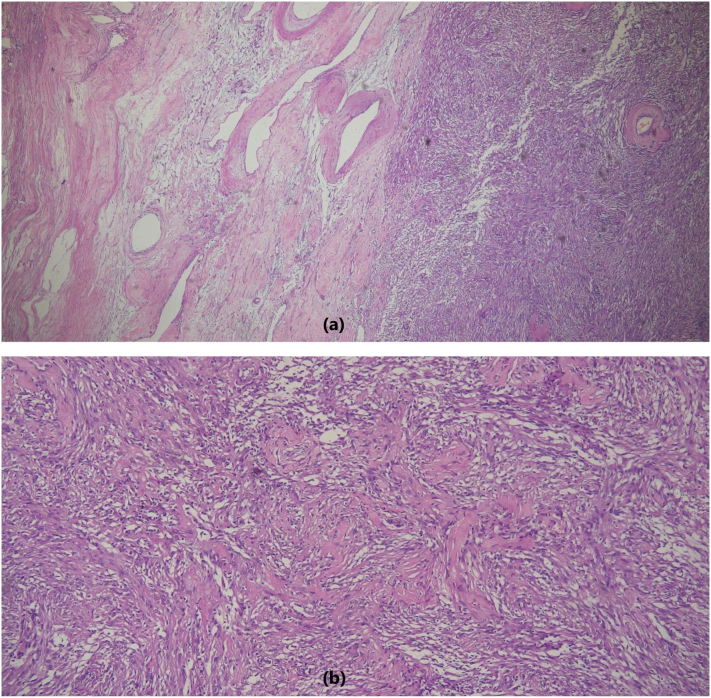
(a): Adrenal schwannoma (H&E, ×40 magnification) showing a well-encapsulated tumor with spindle-shaped neoplasm in the adrenal gland. (b): Verocay bodies with palisading nuclei seen in hypercellular Antoni A areas.

**Fig. 4:**
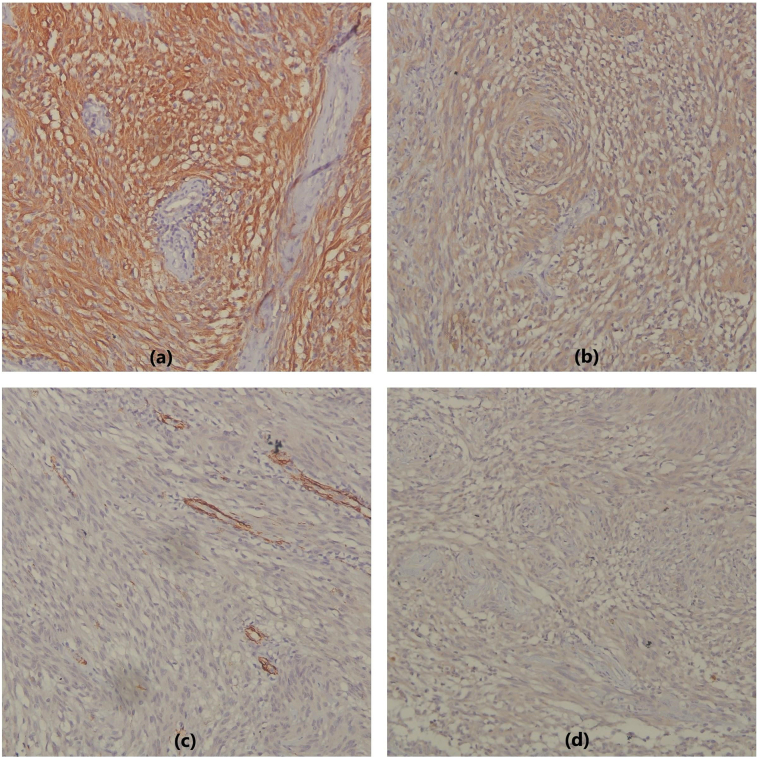
(a): Immunohistochemistry of tumor cells showing strong nuclear and cytoplasmic immunoreactivity for S-100 protein. (b): Immunohistochemistry of tumor cells showing focal weak cytoplasmic desmin immunoreactivity. (c): Immunohistochemistry of tumor cells showing negative immunoreactivity for SMA. (Positive internal control of smooth muscles in the vessel wall). (d): Immunohistochemistry of tumor cells showing focal weak membranous immunoreactivity for CD117.

The resected mass was well-encapsulated which on the cut section showed heterogeneous grey white gelatinous to solid firm areas (Fig. 2a and b). After the radiological and clinical assessment, histopathological examination showed spindle cell tumor composed of hypercellular and hypocellular areas called Antoni A and Antoni B areas respectively (Fig. 3a). Tumor cells in the hypercellular areas have wavy nuclei with pointed tips and show nuclear palisading forming verocay bodies (Fig. 3b) whereas the hypocellular areas showed ovoid to round nuclei with myxoid stroma and variable-sized hyalinized blood vessels. The tumor cells showed strong nuclear and cytoplasmic reactivity for S-100 which further confirmed the diagnosis of adrenal schwannoma. These cells also showed focal weak cytoplasmic positivity for desmin and CD117 while CD34 and SMA were negative. Ki67 proliferative index was very weak (0%) (Fig. 4a, b, c and d).

The post-operative period was uneventful and the patient recovered well. The patient was discharged on the sixth postoperative day. During discharge, all the vitals of the patient were stable and urine output was normal. The patient is on follow-up, doing well and satisfied with the treatment.

## 3 Discussion

Schwannoma arises from Schwann cells encapsulating peripheral nerves of mainly head and neck or peripheral extremities. Retroperitoneal schwannoma is found incidentally using imaging techniques as spacious space allows growing tumors of large size. These are generally asymptomatic [8]. Schwannomas are mostly seen in patients with neurofibromatosis, Von Recklinghausen's disease, and radiation exposure [9]. Our patient had no such significant past medical history of exposure and the tumor was an incidental finding.

A case series of 33 patients showed a median age of diagnosis to be 49 years ranging from 14 to 89 years predominantly in females (male:female = 1:1.2). In this case series, Adrenal schwannoma was found incidentally in 20 (60.6%) patients while 13 (39.4%) patients were symptomatic with symptoms like abdominal pain or discomfort or pain in the flank region. Only one case showed high levels of urinary catecholamines [4]. In contrast, our patient was a 55-year-old male who presented with non-specific abdominal pain.

CT scan of abdomen and pelvis shows adrenal schwannoma usually as heterogeneous, round or oval, well-circumcised mass along with the cystic lesion and occasional calcification and septation [10]. A study by Shen et al. addressed that schwannoma can be ruled out through the arterial phase of CT scan showing necrosis and minimal degree of tumor enhancement [11]. Adrenal schwannomas present as firm, round, well-circumscribed mass with tan-yellow to greyish-white appearance grossly. Most masses are solid and homogeneous in consistency while few are cystic with areas of calcification or hemorrhage [12,14]. The histology of schwannoma shows spindle-shaped cells having elongated to wavy nuclei with alternating hypercellular and hypocellular regions called Antoni A and Antoni B respectively [13]. Our case also had similar histopathological findings. Immunohistochemically, schwannomas have positive S-100 antibodies with negative cytokeratins, CD34, and inhibins that play a pivotal role in the definitive diagnosis [14]. In our case, S-100 was positive while desmin and CD117 were found to be weakly positive.

Schwannomas have non-specific or uncommon findings on different imaging techniques. This makes preoperative diagnosis and ruling out other adrenal masses especially related to retroperitoneum challenging [15]. In our case, there was an adrenal mass suspicious of malignant pathology through USG and CECT. The levels of VMA and metanephrine in urine were measured to rule out other differentials. Pheochromocytoma, myelolipomas, neuroblastoma, ganglioneuroma, cysts, adrenal adenoma, cortical carcinomas, and metastases in other areas are the differential diagnosis for the suspected adrenal mass [4].

Surgery either open or laparoscopic is the mainstay for the treatment of adrenal tumors. A study by Hobart et al. showed no differences in outcomes of the above two surgical techniques. Laparoscopic surgery is the preferred treatment however it remains controversial for the resection of large tumors (>5 cm) [4,16]. Our patient had undergone laparoscopic surgery which was converted to open surgery due to intra-operative complications.

The surgical resection of the tumor has a good prognosis. Zhou et al. concluded that the patient had favorable survival with no evidence of recurrences after follow-up from 7 to 115 months. In contrast, 33–50% of cases have reported having local recurrences in other studies [17]. Our patient had a favorable outcome after surgery. He is doing well now with no other associated symptoms and signs of local recurrences and has been called for follow-up after six months.

## 4 Conclusion

Adrenal schwannomas are rare tumors that are mostly found incidentally and its suspicion could be considered in any adrenal mass. Pre-operative investigations are likely to be inconclusive which can change the outcome of the patient. Thus, prompt surgical excision along with the appropriate histological evaluation and immunohistochemical studies of the mass is crucial for accurate diagnosis and to predict the outcome.

## Consent for publication

Written informed consent was obtained from the patient for publication of this case report and accompanying images. A copy of the written consent is available for review by the Editor-in-Chief of this journal on request.

## Note

No patient and author details are included in the figure.

## Provenance and peer review

Not commissioned, externally peer-reviewed.

## Ethical approval

Not required.

## Funding

None.

## Guarantor

Sujan Timilsina accepts full responsibility for the work and/or the conduct of the study, had access to the data, and control the decision to publish.

## Research registration number

Not applicable.

## CRediT authorship contribution statement

Purushottam Parajuli (PP), Durga Pandit (DP), Shovana Karki (SK), Sansar Babu Tiwari (SBT) = Study concept, Data collection, and surgical therapy for the patient
Sujan Timilsina (ST), Sujan Sharma (SS), and Surya Prakash Joshi (SPJ) = Writing - original draft preparation
Sujan Timilsina (ST), Sanjeev Kharel (SK) and Sansar Babu Tiwari (SBT) = Editing and writing
Durga Pandit (DP), Purushottam Parajuli (PP) = Senior author and manuscript reviewer

All the authors read and approved the final manuscript.

## Declaration of competing interest

None.

## References

[1] Behrend M., Kaaden S., Von Wasielewski R., Frericks B. (2003). Benign retroperitoneal schwannoma mimicking an adrenal mass. Surg. Laparosc. Endosc. Percutan. Tech..

[2] Quayle FJ, Spitler JA, Pierce RA, Lairmore TC, Moley JF, Brunt LM. Needle biopsy of incidentally discovered adrenal masses is rarely informative and potentially hazardous. Surgery 2007;142:497–502; discussion 502–4. 10.1016/j.surg.2007.07.013 17950341

[3] Cury J., Coelho R.F., Srougi M. (2007). Retroperitoneal schwannoma: case series and literature review. Clinics.

[4] Mohiuddin Y., Gilliland M.G.F. (2013). Adrenal schwannoma: a rare type of adrenal incidentaloma. Arch. Pathol. Lab. Med..

[5] Gubbay A.D., Moschilla G., Gray B.N., Thompson I. (1995). Retroperitoneal schwannoma: a case series and review. Aust. N. Z. J. Surg..

[6] Abdessater M., El Mokdad M., Gas J., Sleiman W., Coloby P., Bart S. (2018). Juxta-adrenal schwannoma presenting as a giant adrenal tumor: a case report and a literature review. Int. J. Surg. Case Rep..

[7] Agha R.A., Franchi T., Sohrabi C., Mathew G., Kerwan A., Thoma A., Beamish A.J., Noureldin A., Rao A., Vasudevan B., Challacombe B. (2020 Dec). The SCARE 2020 guideline: updating consensus Surgical CAse REport (SCARE) guidelines. Int. J. Surg..

[8] Theodosopoulos T., Stafyla V.K., Tsiantoula P., Yiallourou A., Marinis A., Kondi-Pafitis A. (2008). Special problems encountering surgical management of large retroperitoneal schwannomas. World J. Surg. Oncol..

[9] International Agency for Research on Cancer, Wiestler O.D. (2016). WHO Classification of Tumours of the Central Nervous System.

[10] Zhang Y.-M., Lei P.-F., Chen M.-N., Lv X.-F., Ling Y.-H., Cai P.-Q. (2016). CT findings of adrenal schwannoma. Clin. Radiol..

[11] Shen Y., Zhong Y., Wang H., Ma L., Wang Y., Zhang K. (2018). MR imaging features of benign retroperitoneal paragangliomas and schwannomas. BMC Neurol..

[12] Kumar S, Karthikeyan VS, Manohar CS, Sreelakshmi K, Shivalingaiah M. Adrenal schwannoma: a rare incidentaloma. J. Clin. Diagn. Res. 2016;10:PD01–2. 10.7860/JCDR/2016/20405.8265 PMC5028547 27656499

[13] Jakowski J.D., Wakely P.E., Jimenez R.E. (2008). An uncommon type of adrenal incidentaloma: a case report of a schwannoma of the adrenal medulla with cytological, histological, and ultrastructural correlation. Ann. Diagn. Pathol..

[14] Shabana W., Raslan W., Hassan S., Al-Tartir T. (2019). Schwannoma masquerading as adrenocortical tumor: a case report and review of literature. Urol. Case Rep..

[15] Tang W., Yu X.-R., Zhou L.-P., Gao H.-B., Wang Q.-F., Peng W.-J. (2018). Adrenal schwannoma: CT, MR manifestations and pathological correlation. Clin. Hemorheol. Microcirc..

[16] Richter KK, Premkumar R, Yoon H-S, Mercer P. Laparoscopic adrenalectomy for a rare 14-cm adrenal schwannoma. Surg. Laparosc. Endosc. Percutan. Tech. 2011;21:e339–43. 10.1097/SLE.0b013e31823ac4d4 22146188

[17] Jeshtadi A., Govada N., Somalwar S.B., Nagulapally S. (Jul 31 2014). Schwannoma of the adrenal gland. J. Med. Allied Sci..

